# The positive effect of eugenol on acute pancreatic tissue injury: a rat experimental model

**DOI:** 10.11604/pamj.2021.38.132.20202

**Published:** 2021-02-05

**Authors:** Alexandra Tsaroucha, Vasileios Kaldis, Michail Vailas, Dimitrios Schizas, Maria Lambropoulou, Apostolos Papalois, Christina Tsigalou, Apostolos Gaitanidis, Michael Pitiakoudis, Constantinos Simopoulos

**Affiliations:** 1 Postgraduate Program in Hepatobiliary/Pancreatic Surgery, Faculty of Medicine, Democritus University of Thrace, Alexandroupolis, Greece,; 2 2nd Department of Surgery and Laboratory of Experimental Surgery, Faculty of Medicine, Democritus University of Thrace, Alexandroupolis, Greece,; 3 First Department of Surgery, National and Kapodistrian University of Athens, Athens, Greece,; 4 Laboratory of Histology-Embryology, Faculty of Medicine, Democritus University of Thrace, Alexandroupolis, Greece,; 5 Experimental-Research Department, ELPEN Pharmaceuticals, Pikermi, Attica, Greece,; 6 Laboratory of Microbiology, Faculty of Medicine, Democritus University of Thrace, Alexandroupolis, Greece

**Keywords:** Acute pancreatitis, eugenol, histopathological evaluation, immunohistochemistry, rat experimental model

## Abstract

**Introduction:**

we present a rat experimental model used to evaluate the possible reduction in the extent of pancreatic tissue injury in acute pancreatitis cases, after administration of eugenol.

**Methods:**

one hundred and twenty Wistar rats were used, which were randomly assigned in 3 groups: sham (n=20), control (n=50) and eugenol (n=50). Acute pancreatitis was induced by biliopancreatic ligation in the control and eugenol groups, but not in the Sham group. In the eugenol group, eugenol was administered per-os. Five histopathological parameters, such as edema, inflammatory infiltration, duct dilatation, hemorrhage and acinar necrosis were evaluated.

**Results:**

at 72 h from acute pancreatitis induction, the total histological score was diminished in the eugenol group (p<0.0005) and duct dilatation and inflammatory infiltration were reduced compared to the control group (p<0.05). In addition, at 72 h, eugenol reduced pancreatic myeloperoxidase activity (p<0.0005).

**Conclusion:**

eugenol, a highly free radical scavenger agent, may have a preventive role in acute pancreatic injury, as it was evident in our rat experimental model.

## Introduction

Acute pancreatitis (AP) accounts nowadays for many incidents, with the large proportion of patients being admitted to the emergency room in a critical condition. Early diagnosis is vital towards the right decision in therapeutic approach, in order to have a good outcome. Despite the fact that clinical research has significantly advanced in recent years, mortality still remains high (10%) [1-4]. The increased mortality rates are due to tissue necrosis (ranging from 7% to 50%, depending on the extent of the necrotic pancreatic tissue), bacterial translocation (which is triplicating the mortality from 10% to 32%) and the presence of pancreatic ascites (which increases the mortality rates from 9% to 36%) [5-11].

As far as the pathophysiology of AP, the cascade of inflammation is thought to begin by intracellular activation of pancreatic proenzymes, which along with enhanced pancreatic duct permeability initiate pancreatic autodigestion. In addition, anti-inflammatory cytokines are produced [9]. Although the inflammatory reaction is localized at the beginning, it may generate a cascade involving significant production of inflammatory mediators and early organ disarrangement, which might evolve into systematic inflammatory response syndrome (SIRS). Complications may include acute respiratory distress syndrome (ARDS), pleural effusions, renal failure and multiple organ dysfunction syndrome (MODS) [12]. The theoretical basis of SIRS and MODS is supported from animal studies [9].

The most common complication in AP and severe acute pancreatitis (SAP) is the acute lung injury that leads to ARDS followed by renal injury [6]. The most important mediators of sepsis that have been studied in the pathogenesis of AP and SAP are tumor necrosis factor alpha (TNF-α), interleukin (IL)-1β, IL-6, platelet activating factor (PAF), IL-10, C5a, reactive oxygen species and reactive nitrogen species [7,8]. It is clear that the cascade of sepsis involving various inflammatory mediators is the culprit to blame for the development of SAP and MOF. Nowadays, there is continuous research to find proper pharmacological treatment suitable to halt the action of the aforementioned anti-inflammatory molecules and arrest the progression of the disease to its severe forms [5,7].

Eugenol (a 4/allyl/2/methoxyphenol) is a food additive or/and drug [13,14], which presents anti-oxidant, anti-inflammatory and deoxyribonucleic acid (DNA) protective properties; this molecule can be found in several plants and fruits [15-17]. A number of experimental models inducing AP in animals have been developed recently [17,18]. A worth mentioning example is the pancreatic duct ligation (PDL) model, that can be implemented in both benign and malignant conditions in human species, simulating effectively the obstruction of ampulla of vater by gallstones [18-20].

The aim of this study is to evaluate the pathologic alterations and adjustments of pancreatic tissue and evaluate the possible diminishment in the degree of acute pancreatic tissue damage after administration of eugenol in a rat experimental model of acute pancreatitis. Furthermore, pancreatic myeloperoxidase (MPO) activity has been thoroughly studied, since it has been shown that it might be used as a marker of neutrophil migration in acute pancreatitis and has been correlated to the severity of pancreatic injury in previously published studies [20].

## Methods

**Experimental animals:** we used 120 male Wistar rats (age: 3-4 months; weighing: 220-350 g). The animals were provided by the Hellenic Pasteur Institute (Athens, Greece) and were maintained in cages with free access to food and water, under standard laboratory conditions (12 h light-dark cycles, 22-25 °C room temperature, and 55-58% humidity). The experimental surgical procedures took place in the experimental facilities of the ELPEN Pharmaceutical Co., Inc., Experimental-Research Center (Pikermi, Attica, Greece). The general handling of the animals conformed to national, European (directive 86/609/EEC) and international guidelines on the protection of animals used for experimental and other scientific purposes, under protocol number K/2284.

**Acute pancreatitis induced experiment model:**the animals were randomly assigned into 3 groups, as described below: sham (open-close laparotomy; n=20), control (duct ligation only, euthanasia at 6, 12, 24, 48 and 72 hours; n=50) and eugenol (duct ligation and administration of eugenol, euthanasia at 6, 12, 24, 48 and 72 hours; n=50). The animals were anaesthetized initially by isoflurane; then, 0.25 mL butorphenol (dolorex; intervet/schering/plough animal health, Boxmeer, Holland) was injected subcutaneously. The animals were then intubated under direct endotracheal laryngoscopy with a 16 G venous catheter, which was then connected to a rodent ventilator (Harvard apparatus, Holliston, MA) set at 70 breaths/min and a tidal volume of 3 mL. The proper intubation was confirmed by the observation of the chest expansion and retraction and lung auscultation. Following intubation, anesthesia was maintained by a mixture of 93% O_2_, 5% CO_2_ and 2% isoflurane.

The animals were subjected to acute pancreatitis as follows [20]: a midline incision was performed to the abdominal wall. The stomach, the small bowel and the pancreas were identified. In the control and eugenol groups, the biliopancreatic duct was ligated close to the duodenal wall with 4-0 silk sutures. The abdomen was then closed. Eugenol in corn oil was administered only to the eugenol group by a nasogastric catheter at a dose of 15 mg/kg. The sham and control groups received only corn oil solution. Butorphenol (2 mL/kg) was used as analgesic after surgery administered subcutaneously. Ketamine (narcetan; vetoquinol, Buckingham, UK) 0.3-0.6 mL and xylazine (rompun; Bayer, Uxbridge, UK) 0.1-0.3 mL were used for euthanasia, which was performed at predetermined times (i.e. 6, 12, 24, 48 and 72 hours postoperatively). Pancreatic tissues were then harvested.

**Preparation of eugenol solution:** pure eugenol (eugenol 99%, Aldrich Chemistry, St. Louis, MO, USA) was purchased and prepared in an oily solution in the chemical laboratory of ELPEN. This was achieved with the admixture of pure eugenol in a corn oil solution at a concentration of 1.5 mg/mL.

**Histopathological evaluation:** the histopathological analysis was carried out at the laboratory of histology and embryology, faculty of medicine, Democritus University of Thrace. Tissue samples were placed in 10% buffered formalin solution, and 4 μm paraffin-embedded sections were stained with hematoxylin/eosin. All specimens were evaluated by a pathologist blinded to the sequence of the biopsy specimens. Slides were evaluated with regard to 5 histopathological parameters, i.e. edema, inflammatory infiltration, duct dilatation, hemorrhage and acinar necrosis [20]. The alterations were quantified according to a scoring system, that ranged from absence to severe lesions (0: none; 1: mild; 2: moderate; 3: severe). The scores of each individual parameter for each slide were added and a histopathological score was obtained for each specimen [20].

**Immunohistochemistry:** tissue sections of 4 μm were mounted on glass slides, dewaxed and rehydrated. The kit, En vision HRP, Mouse/Rabbit detection system (K 5007), DAKO Carpinteria, CA) was used. To inhibit endogenous peroxidase, the specimens were incubated with 3% H_2_O_2_ (200 mL H_2_O and 6 mL H_2_O_2_) for 15 min in a dark room. Before the primary antibody was applied, the sections were immersed in 10 mM citrate buffer (pH 6.0), rinsed in tris-buffered saline and subsequently heated in a microwave oven (650-800 W) for three cycles of 5 min. The slides were washed with tris-buffered saline before application of the primary antibody in order to reduce nonspecific binding of antisera. Control slides were used as common negative controls for all antibody staining. Sections were then briefly counterstained with Mayer´s hematoxylin, mounted and examined under a Nikon Eclipse 50i microscope (Nikon Instruments Inc., NY, USA).

Scoring was assigned according to the proportion of cells with cytoplasmic staining. Positive expression was determined by the number of stained cells. The average labeling index was assessed according to the proportion of positive cells, after scanning the entire section of the specimen. Sections with greater than 10% stained cells were considered as being positive. The results were graded as negative (0) for <10% of cells stained, low (1) for >10% and <30% cells stained, moderate (2) for >30% and <70% cells stained and of high expression (3) for >70% cells stained.

**Determination of pancreatic MPO activity:** the antibody MPO used was myeloperoxidase (rabbit polyclonal), DAKO (A 0398, Carpinteria, CA), that was diluted at 1: 400. The pancreatic tissue was homogenized in 50 mmol/L potassium phosphate buffer (pH 6) with 0.5% hexadecyl-trimethyl ammonium bromide. The homogenates were then centrifuged for ten minutes at 40.000, rpm at 4°C. The resultant supernatant reacted with o-dianisidine dihydrochloride and H_2_O_2_ and the absorbance was determined spectrophotometrically at 450 nm (and 540 nm as control wavelength). MPO activity serves as a marker for neutrophil infiltration [19].

**Statistical analysis:** data were expressed as mean ± standard deviation (SD) for all inflammatory markers. The ordinal nature of inflammatory markers and the violation of normality in distribution lead us to use non-parametric statistical tests. The comparison of variables among the 3 groups was performed using the Kruskal-Wallis test. Pairwise comparisons were done using the Mann-Whitney test. All tests were applied at each time point (6, 12, 24, 48 and 72 hours after the induction of AP). All tests were two-sided, statistical significance was set at p<0.05. All analyses were carried out using the statistical package SPSS version 17.00 (Statistical Package for the Social Sciences, SPSS Inc., Chicago Il., USA).

## Results

**Figure 1:**
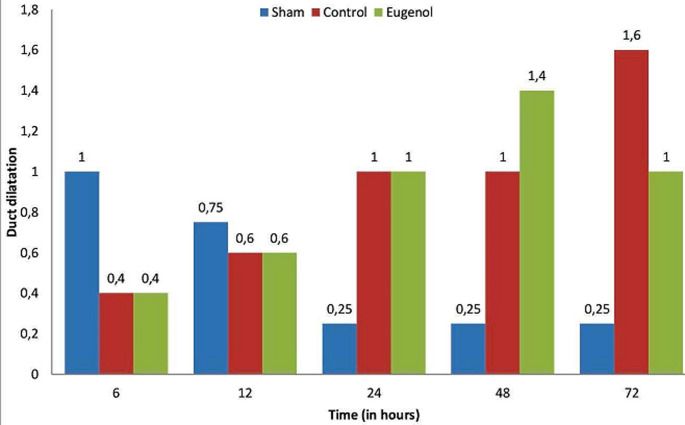
association between duct dilatation and time (0, 6, 12, 24, 48 and 72 h) in sham, control and eugenol group; each column represents mean ± standard deviation; statistical significance was set at p<0.05

**Figure 2:**
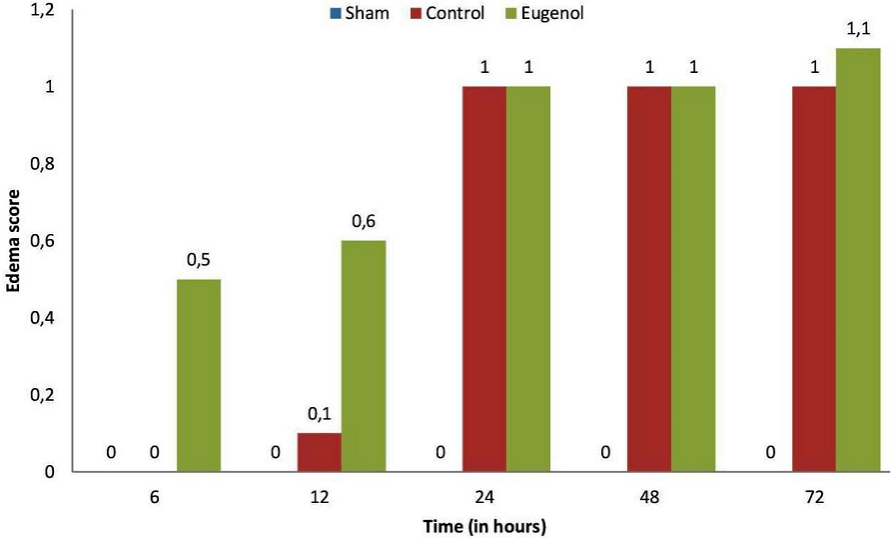
degree of edema score relative to time (0, 6, 12, 24, 48 and 72 h) in sham, control and eugenol group; each column represents mean ± standard deviation; statistical significance was set at p<0.05

**Figure 3:**
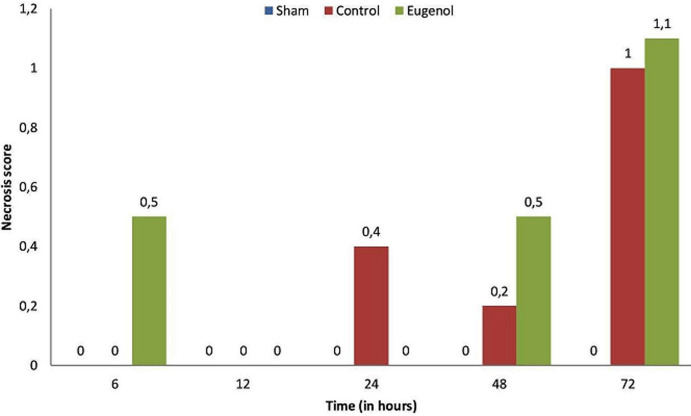
association between necrosis score and time (0, 6, 12, 24, 48 and 72 h) in sham, control and eugenol group; each column represents mean ± standard deviation; statistical significance was set at p<0.05

**Figure 4:**
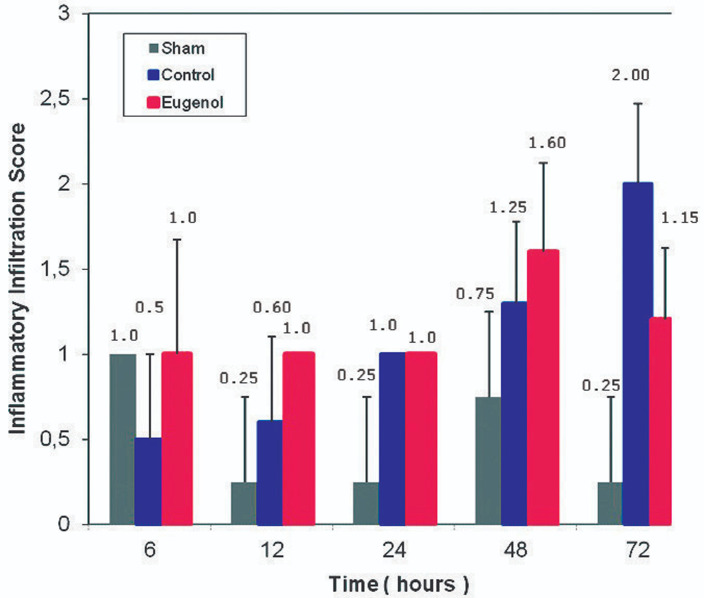
degree of inflammatory infiltration relative to time (0, 6, 12, 24, 48 and 72 h) in sham, control and eugenol group; each column represents mean ± standard deviation; statistical significance was set at p<0.05

Surgical operation was successful and uneventful on all animals, which then resumed normal diet and bowel function. The animals of the sham group showed normal pancreatic parenchyma; no edema, hemorrhage, inflammatory infiltration, duct dilatation or tissue pancreatic necrosis were observed (Figure 1, Figure 2, Figure 3, Figure 4). Animals in the control group showed significant pancreatic parenchyma architectural disturbance, accompanied by severe degree of edema, parenchyma hemorrhage, notable inflammatory infiltration, duct dilatation and pancreatic necrosis. Finally, animals in the eugenol group showed milder morphologic alterations in the pancreatic tissue when compared to the control group (Figure 1, Figure 2, Figure 3, Figure 4).

**Figure 5:**
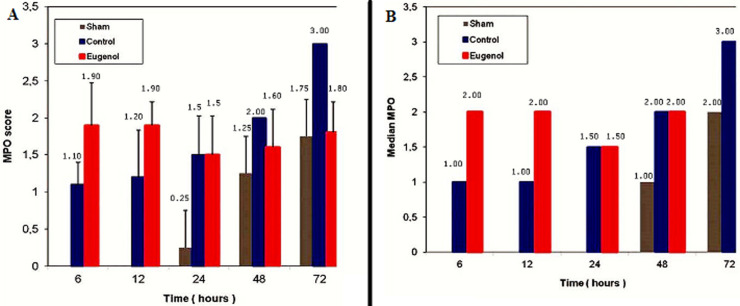
association between: A) myeloperoxidase (MPO) score and time (0, 6, 12, 24, 48 and 72 h); B) median MPO relative to time (0, 6, 12, 24, 48 and 72 h), in sham, control and eugenol groups; each column represents mean ± standard deviation; statistical significance was set at p<0.05

More specifically, as far as hemorrhage is concerned, at the time of sacrifice, the control group showed less hemorrhage than the eugenol group (eugenol versus control group at 12 and 48 hour time points). However, at 72 hours the eugenol group showed no difference when compared to the control group. Regarding duct dilatation in animals with experimental AP, the eugenol group showed no statistical significant difference when compared with the control group at 6, 12, 24, 48 hours postoperatively. On the contrary, at 72 hour (p<0.05), the eugenol group showed less duct dilatation than the control group with statistical significance (Figure 1). In relation to edema and necrosis in animals with experimental AP, the eugenol group showed no difference at the time of sacrifice when compared to the control group (Figure 2, Figure 3). Additionally, with respect to inflammatory infiltration, the Eugenol group showed statistical significant difference at 72 hours, when compared to the control group (Figure 4). Along with this finding, it was evident that eugenol also contributes to the reduction in MPO activity in animals with experimental AP overall from 48 hours (p=0.015) to 72 hours (p<0.0005) (Figure 5).

**Figure 6:**
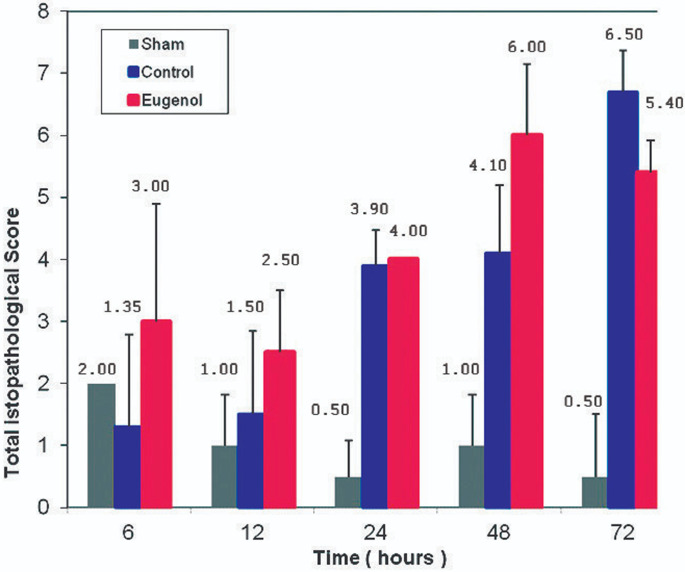
association between the total histopathological score and time (0, 6, 12, 24, 48 and 72 h) in in sham, control and eugenol group; each column represents mean ± standard deviation; statistical significance was set at p<0.05

The total histopathological score for control and eugenol groups was higher in comparison with the sham group at 72 hours and for the whole sample. Eugenol administration resulted in a lower histopathological score in rats with acute pancreatitis (p<0.0005) (Figure 6).

## Discussion

Acute pancreatitis is generally characterized as a condition that exhibits hyperamylasemia, pancreatic interstitial edema, acinar cell injury, leukocyte infiltration and hemorrhage. It occurs because of early activation of proteolytic zymogens within the exocrine pancreas leading to autodigestion of the gland [20,21]. Subsequently, the pro-inflammatory transcription factor nuclear factor- κB (NF- κB) is responsible, when activated, for the substantial increase in expression of various cytokines and mediators. Eventually, anti-inflammatory cytokines are produced leading to a compensatory response syndrome (CARS) [4,9,22,23].

It has been described in several AP published studies that high levels in serum concentrations of the anti-inflammatory mediators, such as TNF-α receptors, IL-10 and IL-11 play a significant role during the course of the disease [4,21,22]. In the case when the anti-inflammatory response is sufficient, the patient´s condition improves. In the unfortunate case of insufficient control, a pro-inflammatory burst leads to multiple organ dysfunction syndrome. Along with this, there might be overcompensation of anti-inflammatory reaction that could lead to excessive inhibition of the immune response, rendering the patient susceptible to immunosuppression and infections [9,22,23].

This study was undertaken in order to determine whether the administration of eugenol has an advantageous result on the pancreatic inflammation in a rat acute pancreatitis experimental model. The usage of eugenol appeared to ameliorate the severity of acute pancreatitis, as indicated by the milder pancreatic tissue injury. To evaluate the extent of pancreatic injury in this experimental model, we examined histopathological parameters such as edema, inflammatory infiltration, duct dilatation, hemorrhage and acinar necrosis by the use of a semi-quantitative scoring system, as previously mentioned.

Interestingly enough, regarding the histopathological assessment, we concluded that the histological score was diminished in the eugenol group experimental model at 72 hours from the inauguration of the process, presenting a statistically significant difference, although the specific figures were not always favorable. As far as hemorrhage in rats is concerned, it seems that eugenol did not have any effect upon it, whereas it reduced the duct dilatation at 72 hours compared to the control group. On the other hand, inflammatory infiltration, as another indicator of pancreatic tissue injury, improved at 72 hours. Additionally, MPO expression has been shown to be less apparent in eugenol group from 48 hours to 72 hours.

An experimental model of AP which successfully induces the inflammation of the pancreatic tissue caused by biliary obstruction and leads to multiple organ failure resembling human manifestations has been used before [5,24]. Ligation of the common biliopancreatic duct in a rat model resembles obstructive acute pancreatitis in humans, which encompasses several characteristics such as pancreatic necrosis, haemorrhage of the pancreatic parenchyma, white cell infiltration and formation of microthrombi at distant organ sites. Obstruction of the common biliopancreatic duct (CBPD) by gallstones inhibits the efflux of pancreatic enzymes, leading to elevated pressure in the pancreatic tissue and creating bile reflux into the pancreatic duct [20,24].

In previously published work [24], researchers demonstrated that by this model, AP is obvious and present, resulting in histopathological changes in pancreatic tissue. Significant edema, intense infiltration of leukocytes and intra-pancreatic bleeding show the similarity of the histopathology of experimental lesions with moderate-to-severe acute pancreatitis in humans [24,25], indicating that the rat model is proper for further studies of acute pancreatitis. Eugenol (4-allyl-2-methoxyphenol) is the principal chemical compound of clove oil, which is primarily derived from a variety of plants like *Eugenia caryophyllus* and *Myristica fragrans*. Researchers point out its anti-inflammatory, antioxidant, anticancer activity, anesthetic and muscle relaxant abilities [19,26,27]. Eugenol also exhibits pharmacological effects in almost all human systems, making it very promising [28].

It has been demonstrated that eugenol can be used in treatment of several gram-positive and gram-negative bacteria by inhibiting their action using the agar well diffusion method. Furthermore, it has been shown that the growth of fungal infections like candidiasis may be suppressed by the use of eugenol in in vivo studies [26]. Along with these anti-inflammatory effects, anti-plasmodial, antiviral and anthelmintic properties have been linked with this compound. The anti-inflammatory activity of eugenol is thought to be linked to its ability to inhibit cyclooxygenases (COX) 1 and 2 [19,26]. Lately, in vitro studies have shown that eugenol has antimitotic effects and can halt the cell viability of HeLa cells in combination with chemotherapeutic drugs like gemcitabine. Interestingly enough, previous experimental studies reported also that eugenol may inhibit the proliferation of melanoma cancer cells in a B16 xenograft model and delay the cell cycle progression in epidermoid carcinoma A431 cells [26,27].

The extent of the injury in pancreatic tissue during AP is well correlated with the levels of free radicals generation. Anti-inflammatory activity of eugenol, by inhibiting PGE2, COX2, IL1b, TNF-α and leukocyte migration, is well documented along with the anti-oxidant capacity of eugenol. The latter feature could be explained by the formation of complexes with reduced metals and in concordance with the anti-inflammatory activity, which is profound at low concentrations. Taking into consideration eugenol´s aforementioned potentials, this might shed light on the observed improvement in AP [29-33].

Efforts have already been made in experimental animal models, in order to investigate the potential pharmaceutical or phytoceutical properties of eugenol to improve AP. Sowjanya *et al*. [34] have shown the protective role of eugenol in ameliorating the detrimental effects of L-arginine induced AP in experimental animal models. To our knowledge, the current study is the first evaluating the effect of eugenol in AP experimental model induced by pancreatic duct ligation. Despite the fact that there are variations that need to be explained like the fact that tissue edema and necrosis are at some points greater in eugenol group, this experimental study highlights the necessity for further studies to be done in order to clarify the exact role of eugenol in AP. It seems, though, that the effects of eugenol might be dose and time dependent and appropriate adjustments in these parameters may play significant role for universal results to be reported.

## Conclusion

Our attempt focused on demonstrating the advantageous effect that eugenol brings about in AP. Eugenol, a highly free radical scavenger agent, is the main phenolic compound extracted from certain essential oils. As it was evident in our experimental model, eugenol was found to have preventive role in acute pancreatic injury in rats, which presents a promising finding to continue the study with clinical research in humans.

### What is known about this topic

- Several studies have shown that eugenol exhibits anti-inflammatory, antioxidant, anticancer activity, anesthetic and muscle relaxant abilities and also exhibits pharmacological effects in almost all human systems;
- Eugenol seems to have protective role in ameliorating the detrimental effects of L-arginine induced acute pancreatitis in experimental animal models.

### What this study adds

- To our knowledge, the current study is the first evaluating the effect of eugenol in acute pancreatitis experimental model induced by pancreatic duct ligation.
